# Cuproptosis-related lncRNAs predict prognosis and immune response of thyroid carcinoma

**DOI:** 10.3389/fgene.2023.1100909

**Published:** 2023-07-04

**Authors:** Yinli Shi, Pei Sheng, Ming Guo, Kai Chen, Hongguang Zhou, Mianhua Wu, Wenting Li, Bo Li

**Affiliations:** ^1^ The First Clinical Medical College, Nanjing University of Chinese Medicine, Nanjing, China; ^2^ Zhongda Hospital Southeast University, Southeast University, Nanjing, China; ^3^ Jiangsu Collaborative Innovation Center of Traditional Chinese Medicine Prevention and Treatment of Tumor, Nanjing University of Chinese Medicine, Nanjing, China

**Keywords:** cuproptosis-related, lncRNA, thyroid carcinoma, immune response, machine learning

## Abstract

**Objective:** To estimate the survival and prognosis of patients with thyroid carcinoma (THCA) based on the Long non-coding RNA (lncRNA) traits linked to cuproptosis and to investigate the connection between the immunological spectrum of THCA and medication sensitivity.

**Methods:** RNA-Seq data and clinical information for THCA were obtained from the Cancer Genome Atlas (TCGA) and Gene Expression Omnibus (GEO) databases. We built a risk prognosis model by identifying and excluding lncRNAs associated with cuproptosis using Cox regression and LASSO methods. Both possible biological and immune infiltration functions were investigated using Principal Component Analysis (PCA), Gene Ontology (GO), Kyoto Encyclopedia of Genes and Genomes (KEGG), and immunoassays. The sensitivity of the immune response to possible THCA medicines was assessed using ratings for tumor immune dysfunction and exclusion (TIDE) and tumor mutational burden (TMB).

**Results:** Seven cuproptosis-related lncRNAs were used to construct our prognostic prediction model: AC108704.1, DIO3OS, AL157388.1, AL138767.3, STARD13-AS, AC008532.1, and PLBD1-AS1. Using data from TCGA’s training, testing, and all groups, Kaplan-Meier and ROC curves demonstrated this feature’s adequate predictive validity. Different clinical characteristics have varying effects on cuproptosis-related lncRNA risk models. Further analysis of immune cell infiltration and single sample Gene Set Enrichment Analysis (ssGSEA) supported the possibility that cuproptosis-associated lncRNAs and THCA tumor immunity were closely connected. Significantly, individuals with THCA showed a considerable decline in survival owing to the superposition effect of patients in the high-risk category and high TMB. Additionally, the low-risk group had a higher TIDE score compared with the high-risk group, indicating that these patients had suboptimal immune checkpoint blocking responses. To ensure the accuracy and reliability of our results, we further verified them using several GEO databases.

**Conclusion:** The clinical and risk aspects of cuproptosis-related lncRNAs may aid in determining the prognosis of patients with THCA and improving therapeutic choices.

## 1 Introduction

Thyroid carcinoma (THCA), a prevalent malignancy of the endocrine system, can be classified based on its origin and degree of differentiation. This includes papillary thyroid carcinoma, follicular thyroid carcinoma, medullary thyroid carcinoma, poorly differentiated thyroid carcinoma, and anaplastic thyroid cancer (Haddad et al., 2022). Differentiated thyroid carcinomas encompass papillary, follicular, medullary, and mixed carcinomas. Papillary thyroid carcinoma is the most predominant subtype of THCA, constituting over 90% of all cases (Cabanillas et al., 2016). THCA is becoming more prevalent worldwide (La Vecchia et al., 2015). An epidemiological study revealed that THCA has grown to the ninth largest incidence of cancer in the population, the incidence rate is rising at a rate of 3%, and the incidence in women is three times higher than that in men (Bray et al., 2018).

Clinical manifestations of THCA may include the development of abnormal thyroid tumors, varying degrees of dyspnea, dysphagia, hoarseness, lymphadenopathy, and cervical pain. Treatment modalities for THCA typically involve surgical intervention, iodine radiation therapy, and thyroid hormone suppression therapy (Cabanillas et al., 2016). The detection rate of THCA, which has low invasion, slow growth, and low mortality, has increased year by year with the advancement of medical technology and social and economic development. Risk factors linked to the development of THCA include exposure to excessive radiation, abnormalities in glycolipid metabolism, fluctuations in thyroid hormone levels, unhealthy lifestyle choices, hereditary factors, and other variables (Fiore and Vitti, 2012; Borena et al., 2014; Albi et al., 2017). Studies have revealed an increase in morbidity and mortality in advanced THCA, and long-distance cancer cell dissemination can severely damage patients’ quality of life and health (Im et al., 2017). Biomarkers reflecting genetic, protein, and metabolomic alterations have been identified to improve tumor detection, diagnosis, prognosis, and monitoring. Despite this, there has been limited research focused on the relationship between THCA and biomarkers. Investigating THCA biomarkers is crucial for enabling personalized prognosis and treatment.

The role of immunotherapy in the field of oncology is gaining increasing attention from researchers due to its successful treatment of a number of tumor types, leading to a change in the way patients with advanced cancer are treated (Gong et al., 2021). However, due to the high level of malignancy and resistance in THCA, patients have poor prognoses, and viable treatment options are limited. The identification of biomarkers has the ability to both predict the outcome of pharmacological immunotherapy and, to a certain extent, avoid the side effects associated with conventional immunotherapy. Individualized therapy that incorporates anticipated biomarkers and immunotherapy may be a promising treatment approach for patients.

In the human body, copper serves a variety of functions in cells that help maintain cellular homeostasis at low concentrations (Tsvetkov et al., 2022). Studies have demonstrated that the disturbance in the equilibrium of copper homeostasis result in cellular toxicity accumulation, leading to abnormal cell death (Kawahara et al., 2021). Mitochondrial respiration may be necessary for the recently identified copper-dependent regulation of cell death. Copper binds directly to lipids in the tricarboxylic acid (TCA) cycle, causing acylated-protein aggregation and loss of iron-sulfur cluster proteins, which leads to proteinotoxic stress and cell death through various mechanisms, including apoptosis, oxidative stress, and autophagy (Tsvetkov et al., 2022). Tumor development is abnormally correlated with copper and iron levels (Mou et al., 2019). Previous research has demonstrated that ferroptosis is associated with immune cell infiltration in THCA patients, indicating essential genes for developing prognostic models for genetic traits and prognosis prediction (Lin et al., 2021). A novel type of cell death known as cuproptosis has gradually risen to the attention of academics as a potential future research focus due to its effects on cancer and development. According to clinical research, imbalance in copper homeostasis can play a significant role in the development of endometrial cancer (Atakul et al., 2020). Breast cancer stem cells may experience endoplasmic reticulum and oxidative stress, elicit damage-related molecular patterns, and are more susceptible to macrophage phagocytosis, all of which can be caused by the endoplasmic reticulum targeting the copper (II) complex (Kaur et al., 2020). Controlling the breakdown of the copper transport-related protein ATP7A reduces intracellular copper accumulation, prevents excessive oxidative stress and iron death, and decreases colorectal cancer cell growth (Gao et al., 2021). The clinical potential of cuproptosis in cancer is significant. Studies have revealed elevated serum copper levels in THCA patients, indicating a possible link between copper level alterations and the development of THCA (Shen et al., 2015). Additionally, it has been demonstrated that the cuproptosis-related gene FDX1 influences the survival prognosis of THCA patients (Zhang et al., 2022). However, research on cuproptosis is in its early stages, and its exact mechanism of action is not yet fully understood.

**FIGURE 1:**
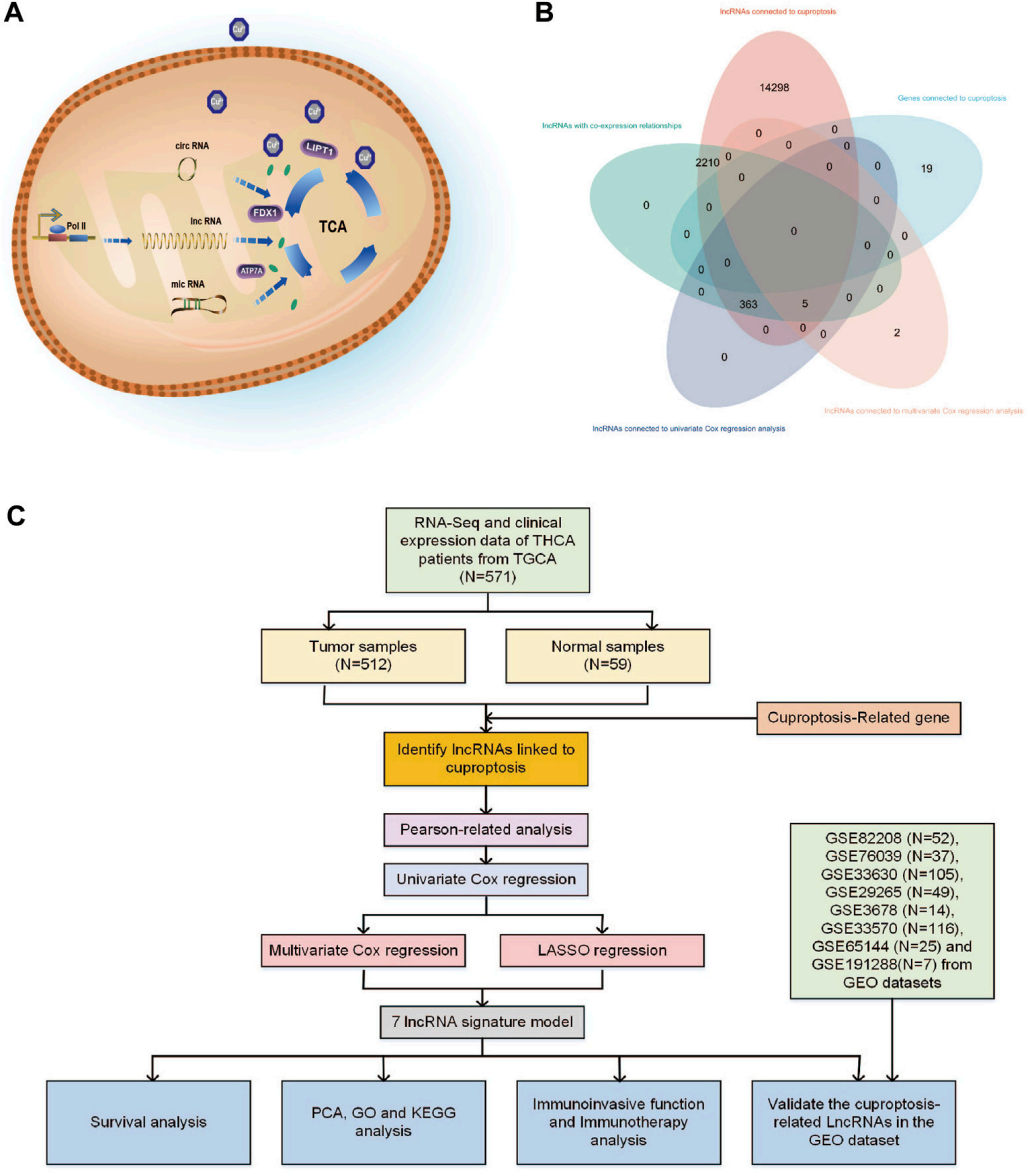
**(A)** Various types of RNA may have different impact mechanisms on copper-induced cell death. **(B)** Venn diagram showing the distribution of lncRNA amount in relation to cuproptosis. **(C)** Flowchart of the bioinformatic study of cuproptosis-related lncRNA on the prognostic function and risk of patients with THCA.

Abnormal copper buildup has the potential to hinder the expression and production of epigenetic elements such as microRNA and lncRNA. Research on cells exposed to copper has revealed that microRNA and circRNA can modulate mitochondrial dysfunction and the TCA cycle, regulate crucial proteins like FDX1, ATP7A, and LIPT1, and alter the mitochondrial membrane potential, as illustrated in Figure 1A (Hsu et al., 2019; Chen et al., 2022; Jin et al., 2022; Liao et al., 2022). Long non-coding RNAs (lncRNAs) are transcripts longer than 200 nucleotides that originate from the genome and exhibit diverse biological activities. As a ceRNA competitive binding microRNA that controls gene expression, it also impacts critical physiological and pathological mechanisms, such as autophagy, development, apoptosis, and cell cycle (Dykes and Emanueli, 2017).

LncRNAs have caught the attention of numerous researchers due to their potential to act as biomarkers and play a multi-functional role in malignancies. LncRNA remains a hot research topic since it is essential for tumor cellular metabolism, apoptosis, invasion, and chemosensitivity (Peng et al., 2017). Furthermore, LncRNAs have the ability to selectively target and control critical proteins involved in immune responses. This can indirectly or directly alter immune cell infiltration in the tumor microenvironment. Overexpression of lncRNA AC003092.1 in glioblastoma has been found to decrease miR-195 function, regulate TFPI-2 expression, improve sensitivity to mortizolomide, and aid tumor cell inhibition (Xu et al., 2018). Expression of lncRNA-NEAT1 predicts prognosis and is an independent predictive factor for overall patient survival in prostate cancer patients (Wen et al., 2020). LncRNA-HOTAIR is extensively expressed in tissues, sera, and cell lines of patients with THCA. Suppression of LncRNA-HOTAIR can prevent tumor cell proliferation, migration, and invasion, ultimately improving patient prognosis (Liu et al., 2020). However, more research is needed to fully understand how lncRNAs affect THCA.

Cuproptosis-related lncRNAs may play a significant role in clinical diagnostic and therapeutic implications for THCA (Zhang et al., 2022). To date, the precise mechanism through which cuproptosis-related lncRNAs affect THCA and predictive models based on cuproptosis genes and immune cells in the THCA’s immune infiltrated microenvironment remain unexplored. To the best of our knowledge, this is the first study to analyze the therapeutic targets linked to THCA from lncRNAs connected to cuproptosis. In this study, we used bioinformatic analysis to analyze cuproptosis-related lncRNAs of THCA patients and their biological and immunological activity and involvement in predicting the prognosis of patients with THCA. Figure 1C depicts the precise operational procedures of this study. The study thoroughly and methodically analyzes the probable link between cuproptosis and lncRNAs in THCA, discusses the potential relevance of cuproptosis-related lncRNAs as prognostic and immunotherapeutic markers for THCA patients, and provides guidance and evidence for the clinical treatment of THCA.

## 2 Materials and methods

### 2.1 Data collection and download for the THCA and GEO transcriptome

The TCGA database (https://portal.gdc.cancer.gov/repository) was utilized in this study to obtain RNA-seq data, clinical data, and mutation data of THCA patients. The dataset contains 512 tumor samples and 59 normal tissue samples. The RNA-seq data were extracted in the fragment per kilobase million (FPKM) format that has been normalized. The log2 (FPKM + 1) transformation was used to normalize the transcriptome data. Related RNA-seq transcriptome and clinical data were extracted, screened, and preprocessed using Strawberry Perl version 5.30.0 (http://www.perl.org). The clinical data were sorted based on age, gender, grade, stage, metastatic/non-metastatic, primary tumor site, and particular tumor site. Eight Gene Expression Omnibus (GEO) datasets (https://www.ncbi.nlm.nih.gov/geo/) were downloaded from GEO: GSE82208 (*n* = 52), GSE76039 (*n* = 37), GSE33630 (*n* = 105), GSE65144 (*n* = 25), GSE29265 (*n* = 49), GSE3678 (*n* = 14), GSE33570 (*n* = 116) and GSE191288 (*n* = 7).

### 2.2 Identification and screening of lncRNAs linked to cuproptosis

Previous studies have provided evidence supporting the role of genes in cuproptosis development (Deigendesch et al., 2018; Tsvetkov et al., 2022). To identify lncRNAs associated with cuproptosis, Pearson correlation analysis was conducted to determine the relationship between the expression levels of cuproptosis-related genes and lncRNA expression, utilizing software packages including “limma,” “dplyr,” “ggalluvial,” and “ggplot2”. The threshold values used were |R|>0.4 and *p* < 0.001. Sankey diagrams were created based on the results of the analysis, illustrating the relationship between cuproptosis genes and their associated lncRNAs.

### 2.3 Construction of prognostic risk assessment model

To develop the best risk and prognosis models, the “glmnet,” “caret,” “survival,” “survminer,” “pheatmap,” and “limma,” packages were used. First, using univariate Cox regression analysis with a threshold setting of *p* < 0.05, the prognostic-associated lncRNA among the cuproptosis-related lncRNAs was found. The results were output in forest plot form. Second, multivariate Cox regression analysis techniques and least absolute shrinkage and selection operator (LASSO) analysis were used to reduce overfitting during modeling. Using 10-fold cross-validation, the best and minimum criteria for the penalty (λ) were chosen. These tools will identify and analyze the lncRNAs of cuproptosis linked to overall survival (OS). The risk score for each patient with THCA was calculated using the following formula:

$$Risk\;score=\sum_{i=1}^{n}\left(Coef\left(i\right)\times \mathrm{Exp}\left(i\right)\right)$$

In the multivariate Cox regression analysis, *n* represents the number of prognostic cuproptosis-related lncRNAs in the risk signature, *Exp(i)* relates to each lncRNA’s expression value, and *Coef(i)* represents each lncRNA’s regression coefficient. The risk score for each sample was computed once the dataset was randomly split into the training and testing groups (Xu et al., 2022). Based on the median risk score, we divided the samples into low- and high-risk groups in TGCA and GEO verification cohorts (GSE29265, GSE33630, GSE65144, GSE76039, GSE82208, GSE3678, GSE33570). Utilizing the “survival” and “survminer” packages, the independent prognostic value of risk prediction variables was evaluated through Kaplan-Meier (KM) curves, which examined the OS and progression-free survival (PFS) of THCA patients across various groups. The “pheatmap” package plots patient survival status and thermal mapping of lncRNA expression depending on risk levels. The training and testing sequence sets were calculated using the Receiver Operating Characteristic (ROC) curve, the Area Under Curve (AUC), and both group series sets under the 1-, 3-, and 5-year areas to evaluate the correctness of the model using the C-index index based on “survival,” “survminer,” “rms,” and “timeROC” packages.

### 2.4 Nomo diagram construction

The risk score was compared across several clinical characteristics. Furthermore, the risk score was integrated with several clinicopathological parameters, and nomo and line plots were constructed to investigate the 1-, 3-, and 5-year survival rates of patients with THCA. The calibration curves were used to compare the expected and actual survival rates.

### 2.5 PCA, GO, and KEGG analysis

PCA was utilized to classify the expression patterns of lncRNAs associated with cuproptosis in THCA samples. The resulting output demonstrated the spatial distribution of high- and low-risk groups as a matrix, which was employed to evaluate the accuracy of the model (Ringnér, 2008). To conduct differential analysis of normalized THCA samples, the “limma” package was used. Multiple test correction was performed using Bayesian technique, and the screening threshold was set to |log2 FC|>1, *p* < 0.05 to identify differentially expressed genes (DEGs). Subsequently, “ggplot2,” “dplyr,” “enrichplot,” “org.Hs.e.g.,.db,” and other packages were downloaded. GO and KEGG pathway enrichment analysis of DEGs was conducted with a P-value threshold of <0.05, and the results were illustrated in the form of a bar graph.

### 2.6 Immunoinvasive function and tumor mutation load analysis

The “estimate” installation package was used to estimate immune and stromal cells in both high- and low-risk groups. The “GSVA,” “limma,” and “GSEABase” packages carried out ssGSEA scoring of infiltrating immune cells and immune-related functions in THCA, and the findings were output in box patterns (Xiao et al., 2020). The CIBERSORT algorithm was utilized to quantify the percentage of immune cells that infiltrate the tumors. The maftools package was used to examine the genetic variance between the two groups by reading the gene mutation file of the high- and low-risk samples and visualizing the 15 genes with the highest mutation frequency. Furthermore, the “ggpubr,” “survival,” and “survminer” packages were utilized to compare the TMB and survival rates between samples from the high- and low-risk groups.

### 2.7 Immunotherapy analysis and drug screening

We analyzed the TIDE database (http://tide.dfci.harvard.edu/login/), which provides information on immunological dysfunction and exclusion caused by THCA. Immunological response has been assessed using well-known immune checkpoints (ICPs). We analyzed potential differences in ICPs responsed between the low- and high-risk groups. (Jiang et al., 2018). Moreover, we used the “pRRophetic” package to predict the IC_50_ values of drugs that could potentially treat THCA in the high- and low-risk groups (Geeleher et al., 2014).

### 2.8 Validation of GEO datasets

independent GEO validation cohorts, the expression levels of various lncRNAs in THCA and non-tumor thyroid samples were evaluated. The “Seurat” package was subsequently utilized and standard downstream processing for scRNA-seq data was conducted (GSE191288). Data normalization was carried out using the NormalizeData function, followed by the extraction of 2,000 genes with high intercellular coefficients of variation. PCA was then performed, with PCs selected for subsequent uniform manifold approximation and projection (UMAP) analysis. Cell types within the resulting clusters were annotated based on the reported cell marker genes.

## 3 Results

### 3.1 Identification of co-expression and predictive significance of cuproptosis-related lncRNA in patients with THCA

**FIGURE 2:**
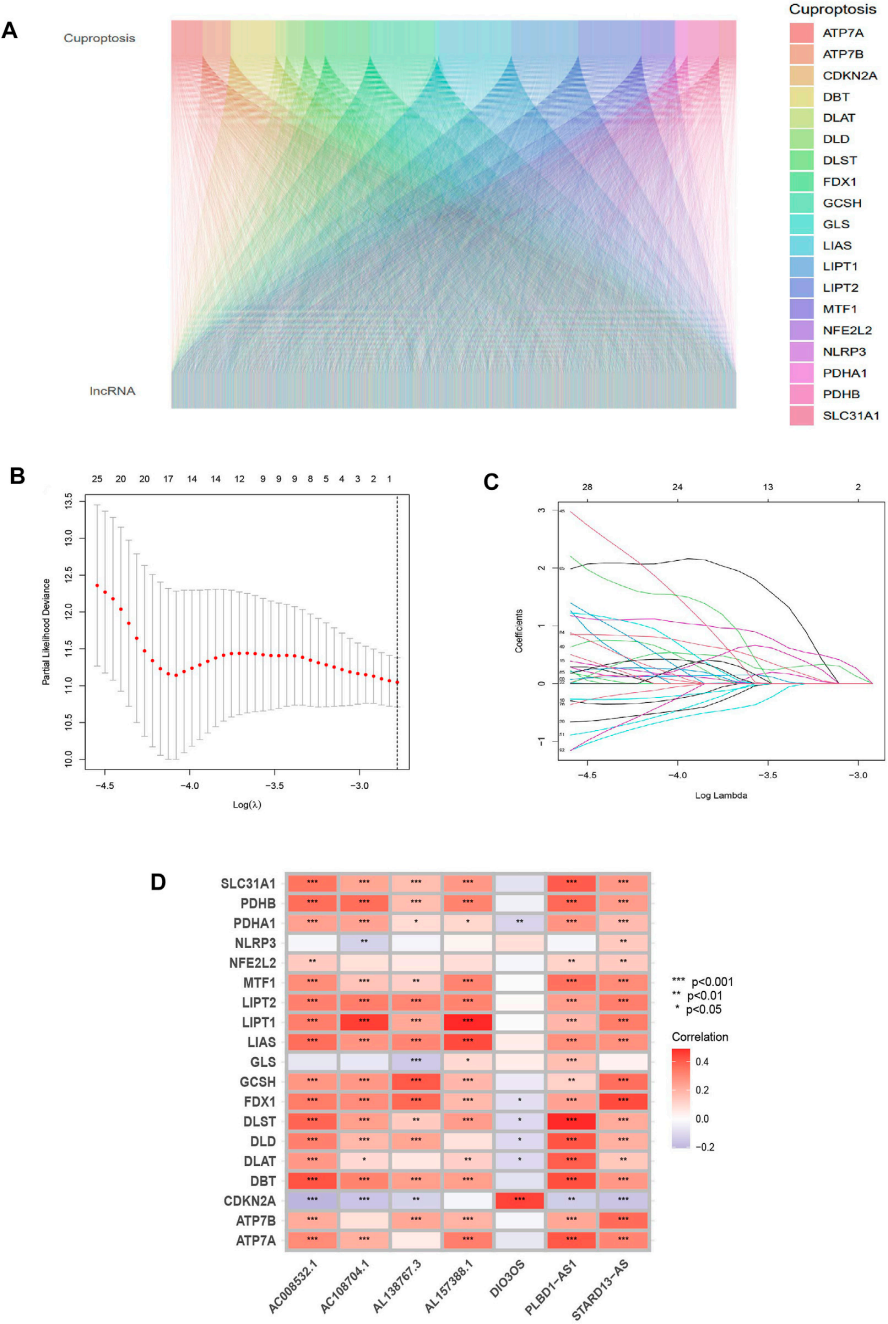
Identifying the predictive features of cuproptosis-related lncRNAs. **(A)** Sankey diagram of cuproptosis-related genes and lncRNAs. **(B)** Distribution of cuproptosis-related lncRNAs’ LASSO coefficients. **(C)** The 10-fold cross-validation of variable selection in the LASSO algorithm. **(D)** Correlation between lncRNAs and genes related to cuproptosis in risk models.

From the TCGA database, 16,877 lncRNAs and 19 genes connected to cuproptosis were retrieved, and co-expression analysis screening was used to identify 2,578 lncRNAs with co-expression relationships. The training (N = 252) and testing (N = 252) groups received 504 patients with THCA in total. Using univariate Cox regression analysis, 368 lncRNAs were identified. The Supplementary Figure S1 contained these data. 252 lncRNAs were linked to cuproptosis using LASSO and Cox regression analysis, and seven lncRNAs were found to be independent prognostic factors using multivariate Cox analysis: AC108704.1, DIO3OS, AL157388.1, AL138767.3, STARD13-AS, AC008532.1, and PLBD1-AS1 (Figure 1B). According to the calculated risk score = AC108704.1 × (−4.6479) + DIO3OS × (0.8558) + AL157388.1 × (3.8603) + AL138767.3 × (0.9341) + STARD13-AS × (4.8836) + AC008532.1 × (5.9321) + PLBD1-AS1 × (−2.1824) (Figure 2).

### 3.2 Establishment and validation of cuproptosis-related lncRNA risk model

**FIGURE 3:**
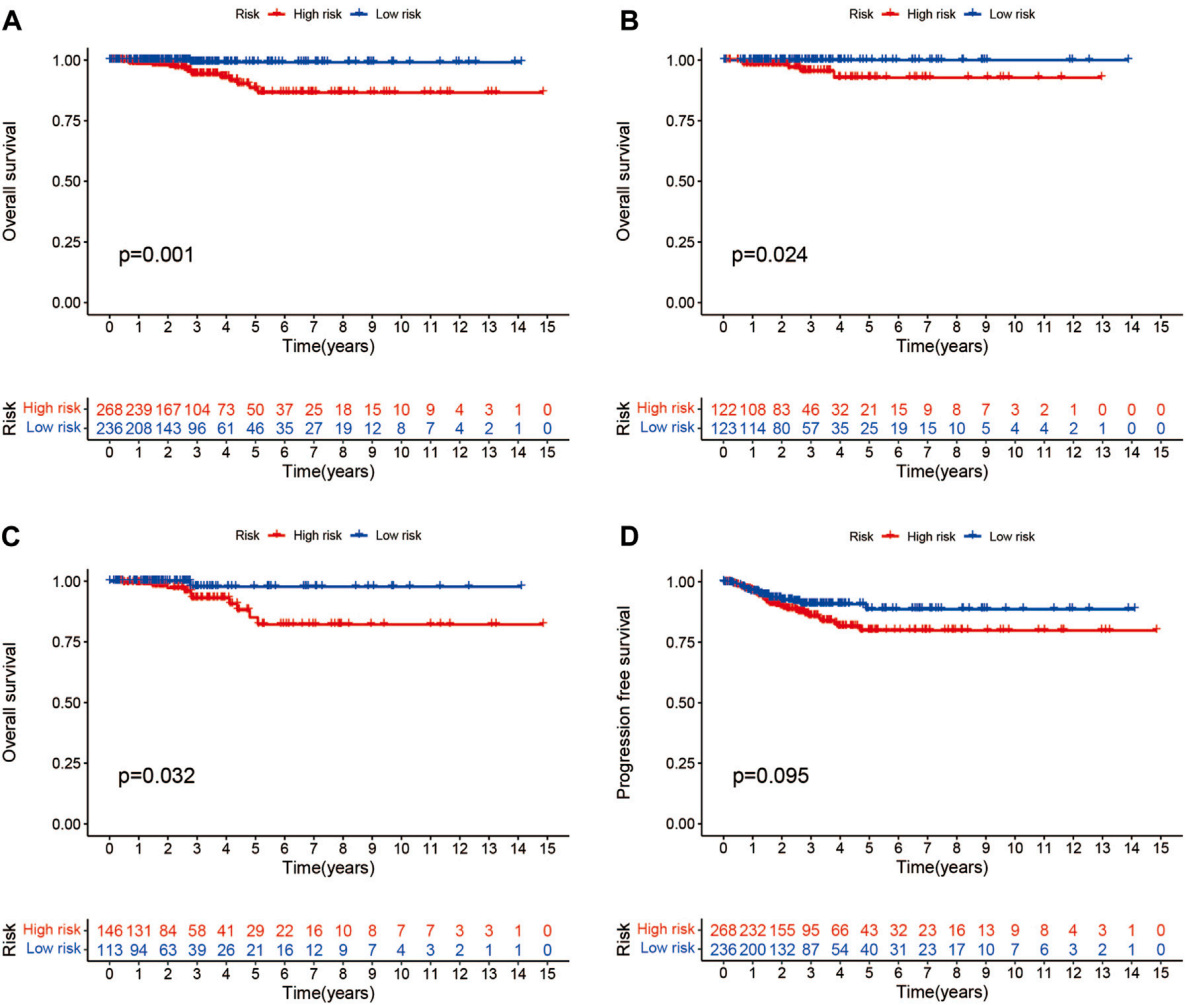
Different groups of patients with THCA survival and progression-free survival under the Kaplan-Meier survival curve differ from one another. **(A–C)** Kaplan-Meier survival curves of patients with THCA for overall, training and testing survival. **(D)** Differences in progression-free survival between high- and low-risk groups.

**FIGURE 4:**
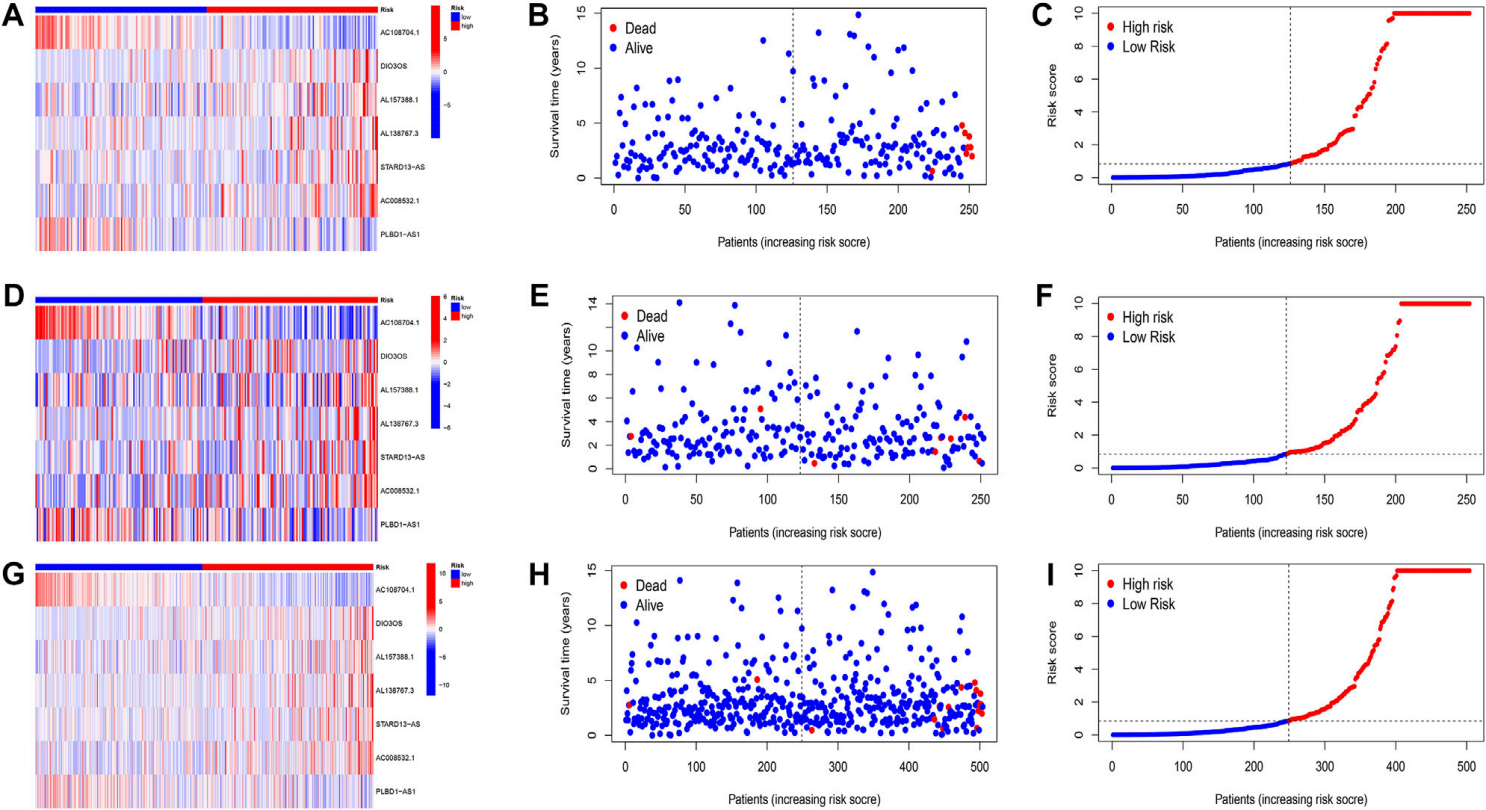
Evaluation of the prognostic signature of cuprotosis-related lncRNAs for accuracy in prognosis prediction. Heatmaps of cuprotosis-related lncRNA expressions **(A,D,G)**, survival time and survival status **(B,E,H)**, and the distribution of overall survival risk scores for training, testing and overall groups **(C,F,I)**.

**FIGURE 5:**
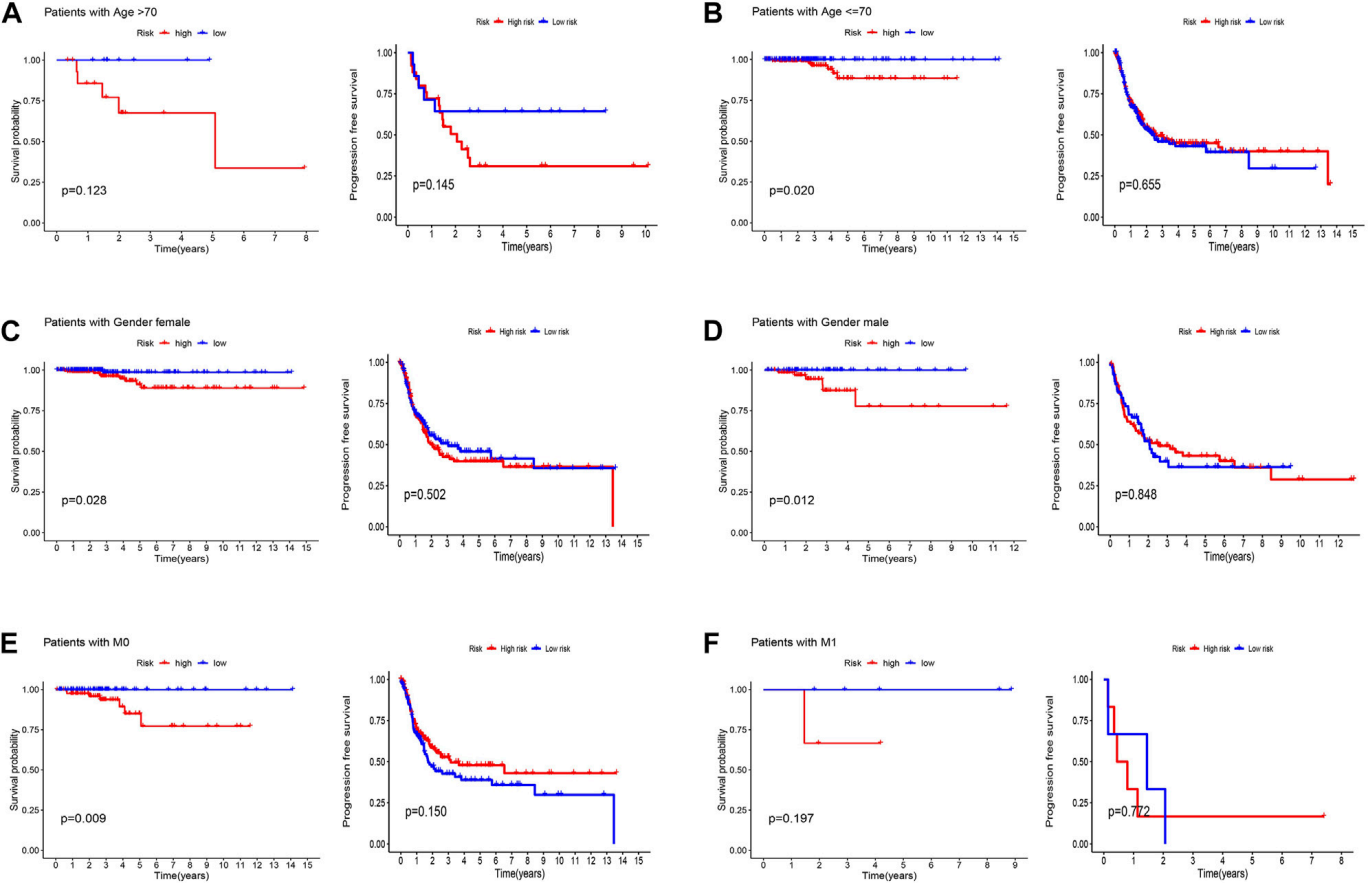
Kaplan-Meier and progression-free survival curves by age **(A,B)**, gender **(C,D)**, and M stage **(E,F)** for low- and high-risk groups.

**FIGURE 6:**
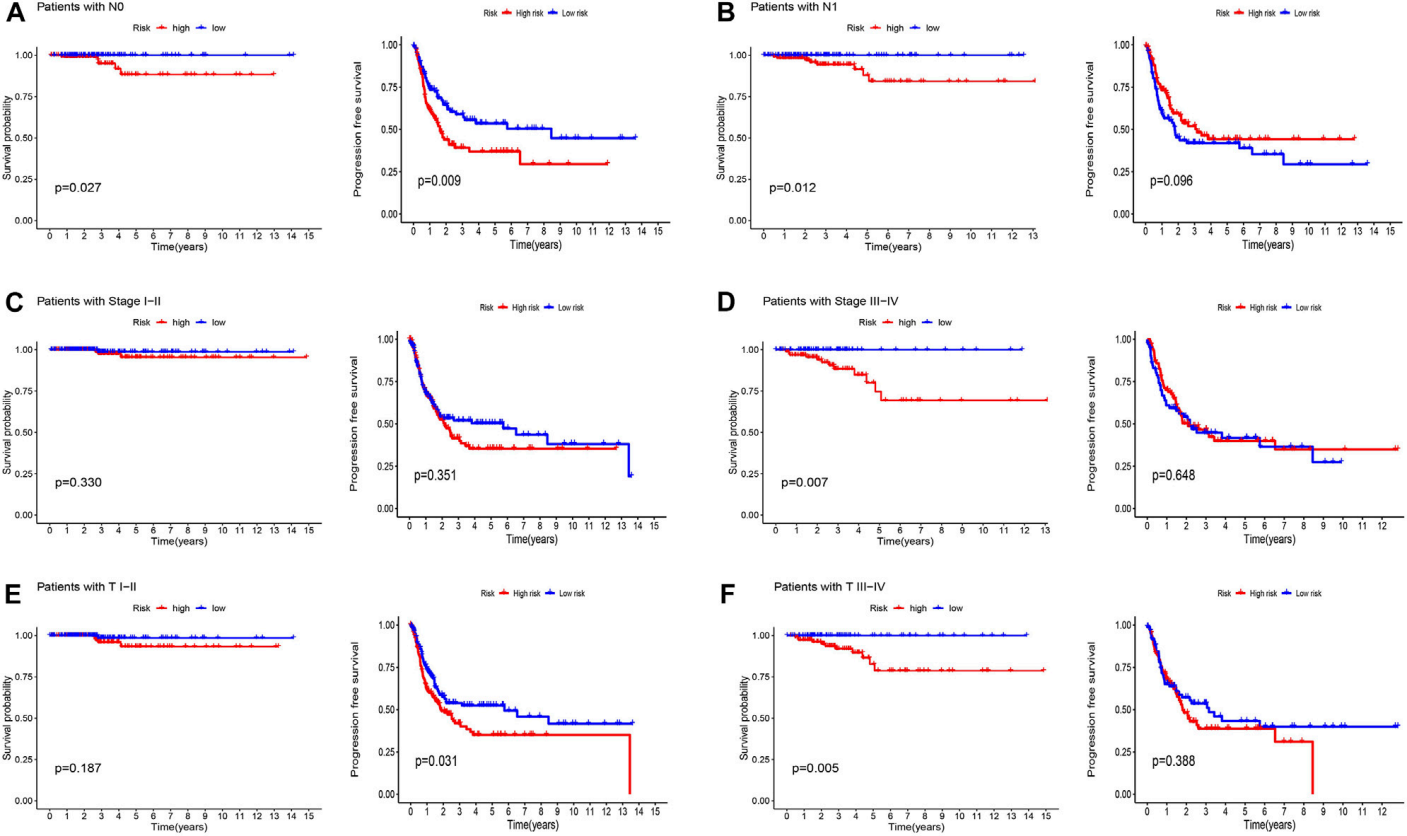
Kaplan-Meier and progression-free survival curves by N stage **(A,B)**, stage **(C,D)**, and T stage **(E,F)** for low- and high-risk groups.

Patients were classified into high- and low-risk groups by computing a risk score based on the median value. The survival of the high-risk group significantly declined over time compared to the low-risk group (*p* < 0.05) in both the training and test groups. Survival analysis revealed that patients from the TCGA verification cohort in the low-risk group had a better prognosis than those in the high-risk group (Figures 3A–C). lncRNA AC108704.1 and PLBD1-AS1 were expressed in the low-risk group, whereas lncRNAs DIO3OS, AL157388.1, AL138767.3, STARD13-AS, and AC008532.1 were expressed in the high-risk group (Figures 4A, D, G). However, no significant difference in PFS was observed between the high- and low-risk groups (*p* > 0.05) (Figure 3D). Patients with THCA were categorized according to age, sex, stage, T phase, M phase, and N-phase in order to ascertain whether the survival rate and risk score were affected by clinical parameters. The results indicated that in the male-female sex group, N0 and N1 subgroups, age ≤70 years subgroup, M0, stage III-IV, and T stage III-IV subgroups, the prognosis for the high-risk group was worse than that of the low-risk group. No significant difference was observed in progression-free survival between the high-risk and low-risk groups with respect to age, sex, stage, TIII-IV, M, and N1 (*p* > 0.05). Nonetheless, a significant difference was identified in TI-II and N0 (*p* < 0.05). The influence of gender on THCA pathogenesis in the high-risk group was not apparent. However, factors such as younger age, tumor metastases, and lymph node invasion were found to affect the prognosis of patients. In the M1 phase subgroup, no significant difference was observed between the high- and low-risk groups. We hypothesize that the lack of significance could be due to the small sample size and poor prognosis of patients with advanced M1 stage THCA (Figures 5, 6).

### 3.3 Construction and analysis of cuproptosis-related lncRNA prognostic model

**FIGURE 7:**
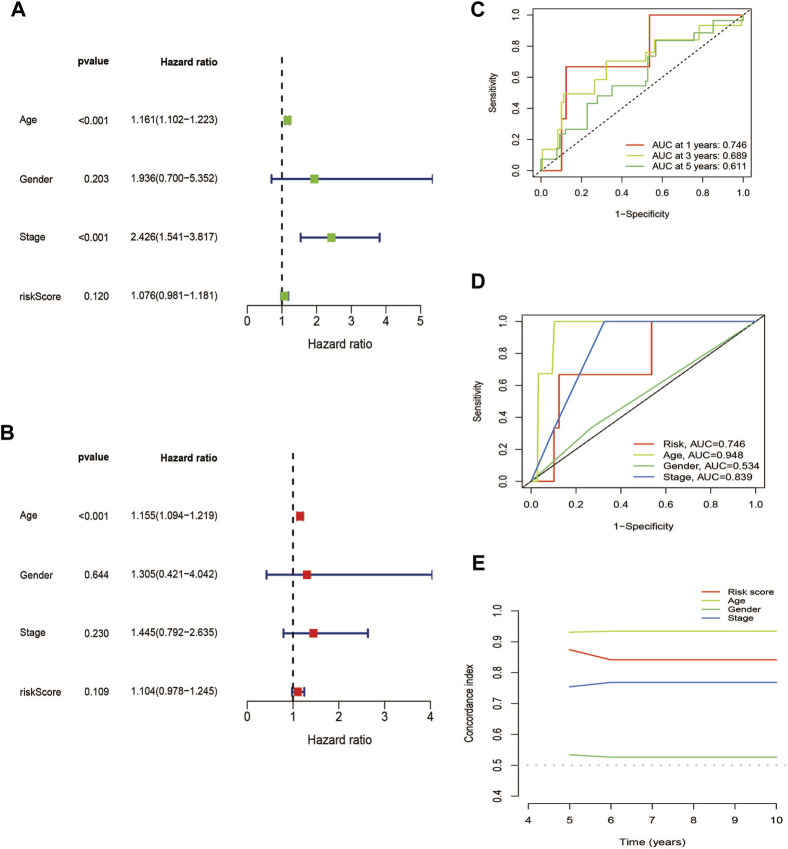
Validation of the cuproptosis-related lncRNAs signature for THCA in TCGA as an independent predictor of outcome. **(A)** Univariate Cox analysis of cuproptosis-related lncRNA prognostic model. **(B)** Multivariate Cox analysis of cuproptosis-related lncRNA prognostic model. **(C)** The prognostic signature’s ROC curve in the THCA sets. **(D)** Under the ROC curve, AUC index of various clinical prognostic characteristics. **(E)** The C-index curves for evaluating the clinical parameters and risk score’s capacity to discriminate at each time point.

The association between patient prognosis and sex (HR, 1.936; *p* = 0.203) was not statistically significant, while age (HR, 1.161; *p* = 0.001) and stage (HR, 2.426; *p* = 0.001) were found to be significant based on the univariate Cox analysis. Multivariate Cox analysis results demonstrated that age (HR, 1.155; *p* = 0.001) was fundamentally related to OS (Figures 7A, B). Supplementary Table S1 provided basic information on patients. Supplementary Figures S2, S3 showed nomo plots and calibration curves to predict 1-, 3-, and 5-year OS of patients with THCA. We developed a line map that precisely predicts 1-, 3-, and 5-year survival in patients with THCA based on prognostic and clinicopathological characteristics of lncRNAs related to cuproptosis. The risk model demonstrated strong stability with AUC values of 0.746, 0.689, and 0.611 for 1-, 3-, and 5-year under the ROC curves, respectively (Figure 7C). The clinical prediction model’s AUC values were 0.746, 0.948, and 0.839 for risk score, age, and stage, respectively, demonstrating high predictive capacity and superior to the AUC values for sex (Figure 7D). Similarly, the C-index also supported the above conclusions (Figure 7E). In summary, we discovered that stage and risk score can be employed as significant clinical prognostic indicators in patients with THCA, and the combined models can be used to aid patient prognoses.

### 3.4 PCA, GO, and KEGG analysis

**FIGURE 8:**
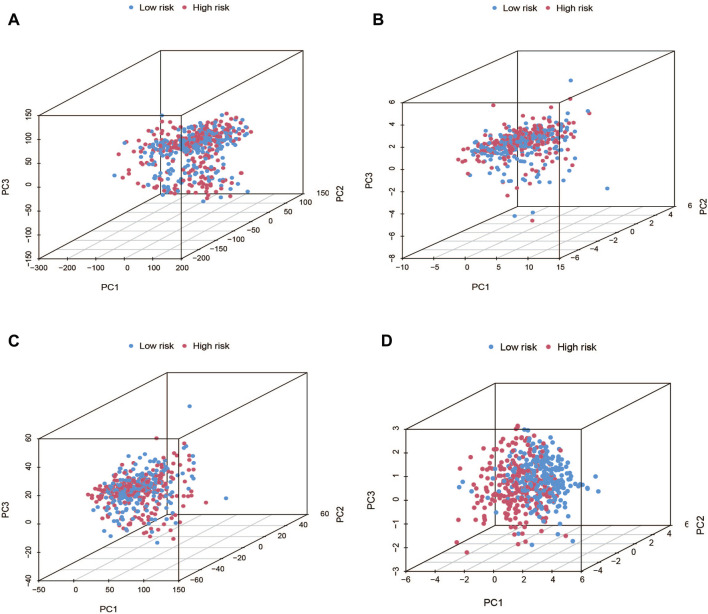
PCA plots showed how samples were distributed according to the expression of **(A)** all genes, **(B)** genes associated to cuproptosis, **(C)** lncRNAs connected to cuproptosis, and **(D)** lncRNAs that increase the risk of cuproptosis.

**FIGURE 9:**
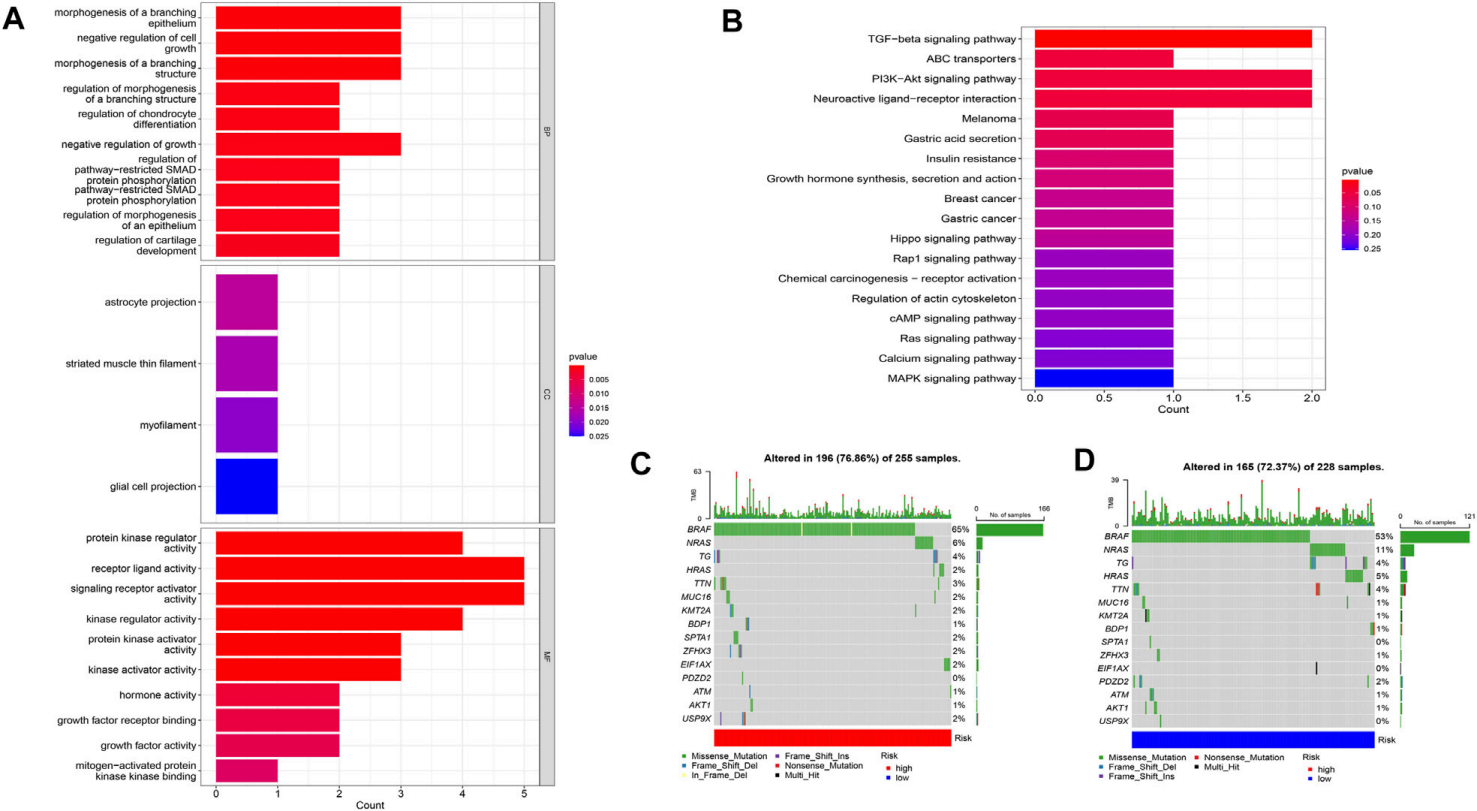
Analysis of GO and KEGG **(A,B)**, as well as waterfall plots of somatic mutation characteristics in high- and low-groups **(C,D)**.

Using PCA, no appreciable changes were found in the expression profiles of all genes, the cuproptosis-related genes, and the lncRNAs associated with cuproptosis. It is easy to distinguish between the low- and high-risk groups using risk models for the expression profile classification of lncRNAs linked to cuproptosis (Figure 8). Eighteen DEGs were identified between the high- and low-risk groups using screening criteria: AC063926.1, TCL1A, NRK, GDF6, AC103563.3, ABCC11, RGS4, FGF7, THRSP, CSMD1, GLTS1, SST, TRIB3, MT3, GREM1, PNOC, TUNAR, TNNI2. GO analysis results revealed that biological processes, including protein synthesis, transport, angiogenesis, apoptosis, enzyme regulation, copper ion binding, and immune response, were all intimately associated with cuproptosis-related lncRNAs. KEGG analysis demonstrated that the TGF-β signaling pathway, PI3K-Akt signaling pathway, neuroactive ligand-receptor interaction, and ATP-binding transporter were enriched in DEGs (Figures 9A, B). These findings suggest that intracellular copper ion concentration may alter the levels of TCA-related metabolites, modify protein synthesis, and impact lipid acylation through signaling pathways related to inflammation and immune function. This induces proteotoxic stress, leading to in-cell death and influencing the progression of the THCA disease.

### 3.5 Immune infiltration analysis

**FIGURE 10:**
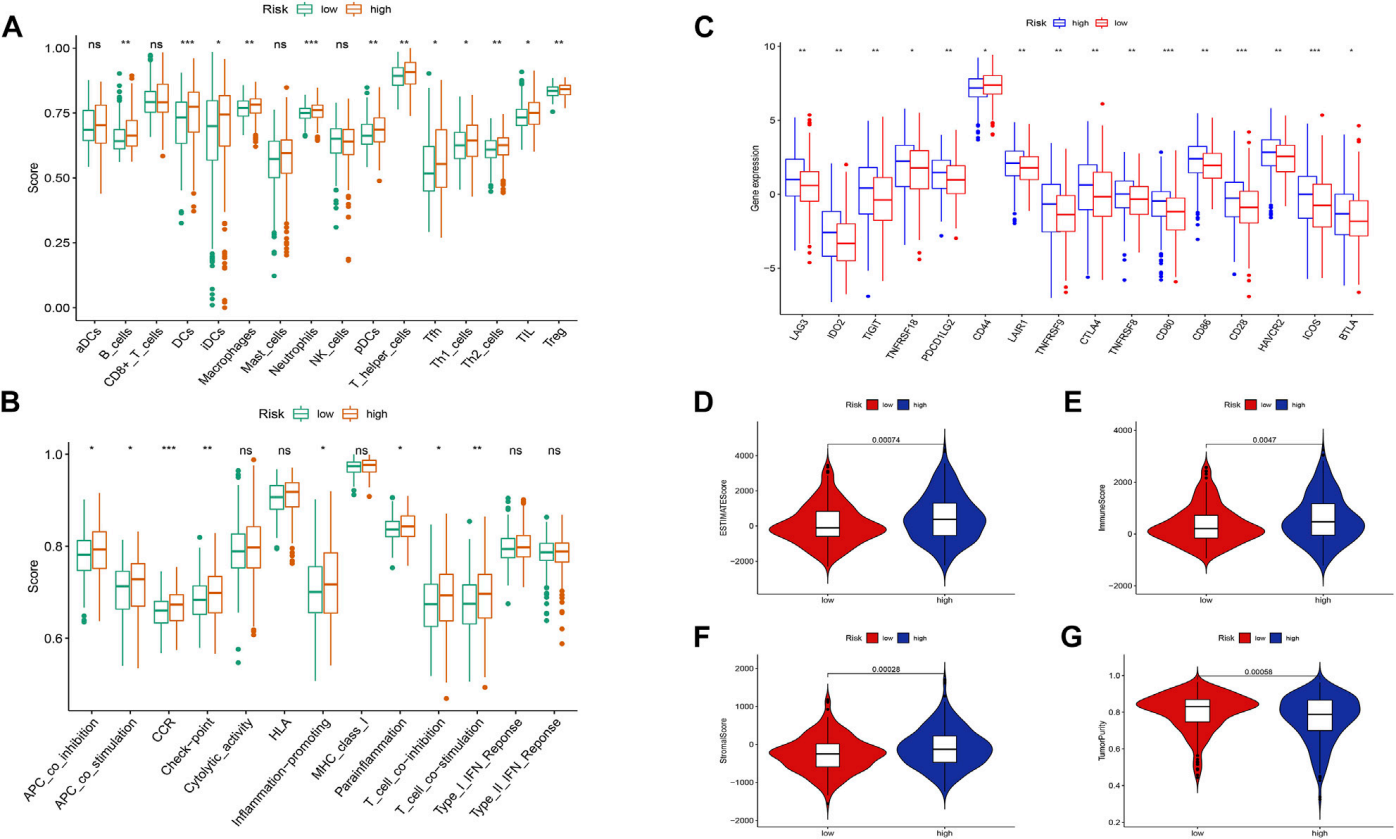
Differences between the low- and high-risk groups in the tumor immune microenvironment. **(A)** The ssGSEA scores of 16 immune cells. **(B)** The ssGSEA scores of 13 immune-related functions. **(C)** Comparison of ICPs between the low- and high-risk groups. Additionally, the box plots comparing the ESTIMATE Score, Immune Score, Stromal Score, Tumor Purity, and between the two risk groups. **(D–G)**. nsP ≥ 0.05, *p < 0.05, **p < 0.01, ***p < 0.001.

Based on the risk scoring algorithm of ssGSEA, we investigated the association between THCA, immune cell infiltration, and immune function, since the tumor immune microenvironment plays a significant role in the formation of malignancies. The high-risk group had significantly lower tumor purity scores but higher ESTIMATE, immune, and stromal scores than the low-risk group (*p* < 0.05) (Figures 10D–G). Inflammatory response, inflammation promotion, ATC co-stimulation, T cell co-stimulation, and cytokine and cytokine receptor (CCR) were significantly different between the high- and low-risk groups in terms of immune-related activities (*p* < 0.05) (Figure 10B). The ratio of B cells, dendritic, macrophages, neutrophils, and T cells were increased in the high-risk group (Figure 10A). Moreover, we analyzed the expression of immunological checkpoint genes in the two groups and found that, with the exception of CD44, genes tended to have higher expression levels in the high-risk group (Figure 10C). Combining the aforementioned findings leads to the conclusion that THCA tumor immunity may be intimately associated with cuproptosis-related lncRNAs.

### 3.6 TMB and TIDE analysis

**FIGURE 11:**
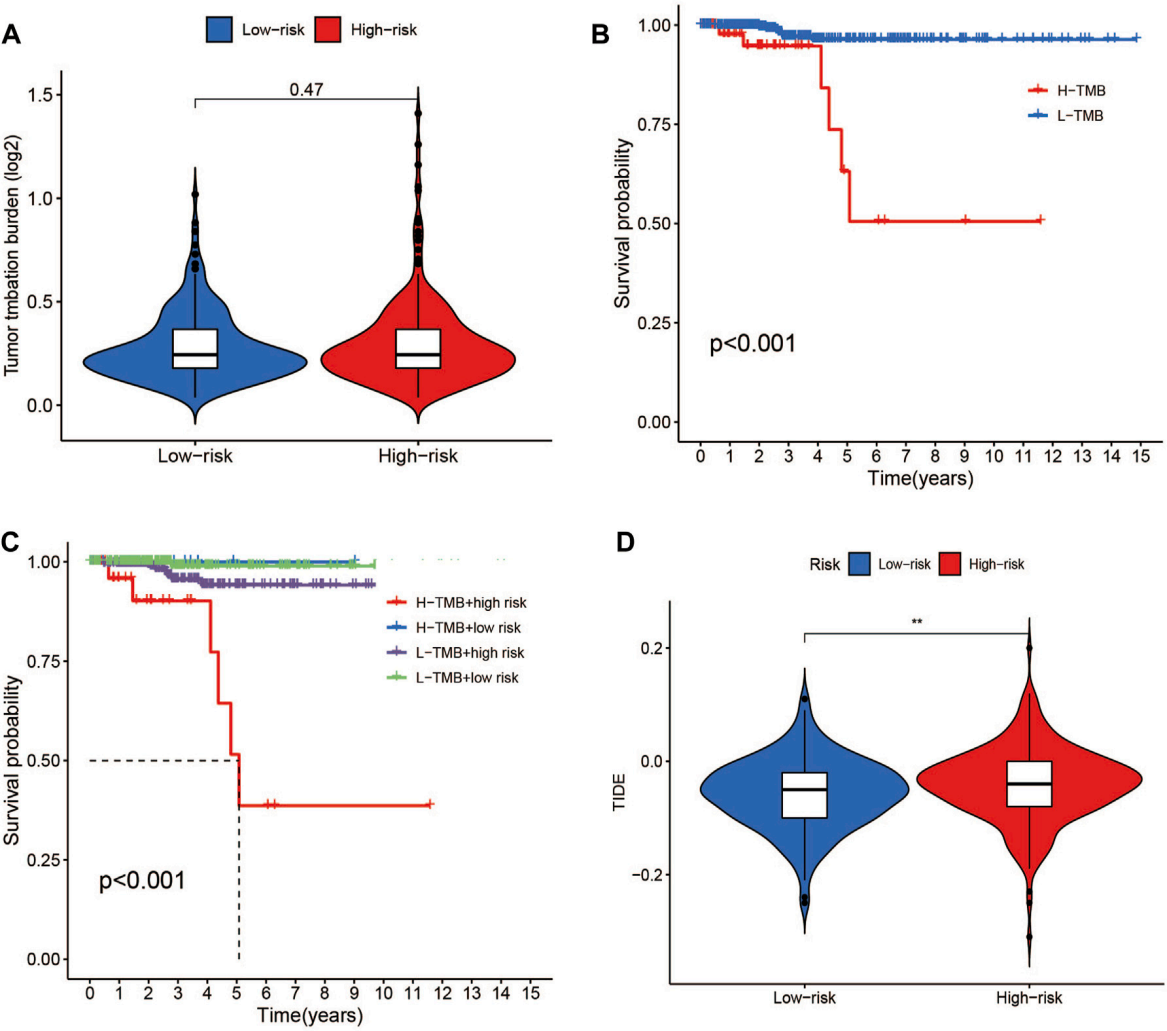
TMB and TIDE analysis. **(A)**TMB between the low- and high-risk groups. **(B)** Kaplan-Meier survival curves for high- and low-TMB groups. **(C)** Kaplan-Meier survival curves by TMB and risk for 4 groups. **(D)** TIDE scores between the low- and high-risk groups. *p < 0.05, **p < 0.01, ***p < 0.001.

Data on the top 15 mutant genes with significant differences between the high- and low-risk groups were obtained from the TCGA database. These genes include BRAF, NRAS, TG, HRAS, TTN, MUC16, KMT2A, BDP1, SPTA1, ZFHX3, EIF1AX, PDZD2, ATM, AKT1, and USP9X, and their analysis results are presented in Figures 9C, D. The analysis of TMB data did not show any significant difference between the high- and low-risk groups (*p* > 0.05) (Figure 11A). However, patients with high TMB had significantly shorter survival and poor prognosis (*p* < 0.05) (Figures 11B, C), and the high-TMB and high-risk groups had the lowest survival prognosis. Furthermore, the high-risk group exhibited a significantly higher TIDE score than the low-risk group (*p* < 0.05) (Figure 11D).

### 3.7 Drug sensitivity analysis

**FIGURE 12:**
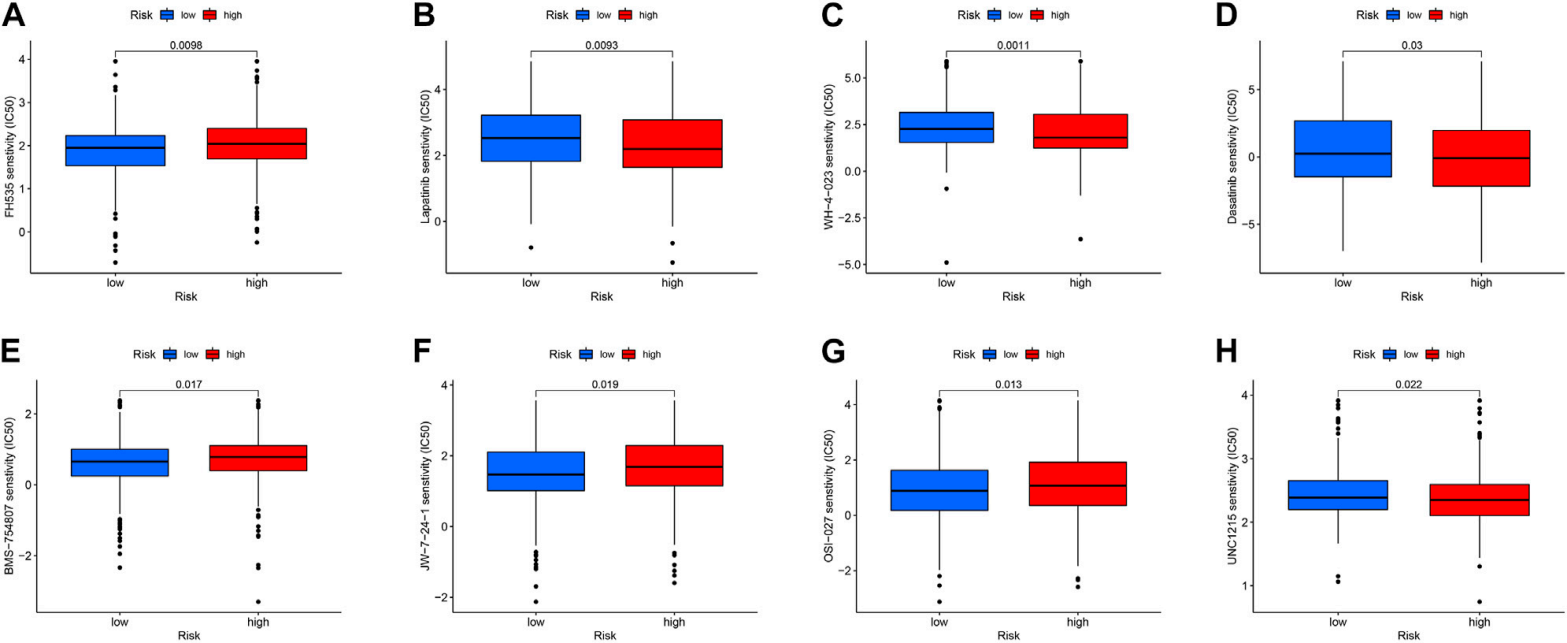
**(A–H)** Drug sensitivity analysis of FH535, Lapatinib, WH-4-023, Dasatinib, BMS-754807, jw-7-24-1, OSI-027, UNC1215.

Targeted drug therapy is another crucial method for treating tumors in addition to immunotherapy. To investigate the relationship between drug sensitivity and the high- and low-risk groups, various anticancer medications were chosen. This research serves as a guide for clinical targeted therapy and future prevention of drug resistance in patients with THCA. The results demonstrated that the sensitivity to WZ-1-84, WH-4-023, UNC1215, lapatinib, GNF-2, dasatinib, and CGP-60474 was significantly higher in patients in the high-risk group compared to those in the low-risk group (*p* < 0.05). Conversely, patients in the low-risk group, including TW37, OSU-03012, OSI-027, KIN001266, JW7241, FH535, and BMS754807, were more sensitive than those in the high-risk group (*p* < 0.05) (Figure 12). These findings can potentially provide insights for future targeted therapy and drug resistance prevention in patients with THCA.

### 3.8 Validate the cuproptosis-related LncRNAs in the GEO dataset

**FIGURE 13:**
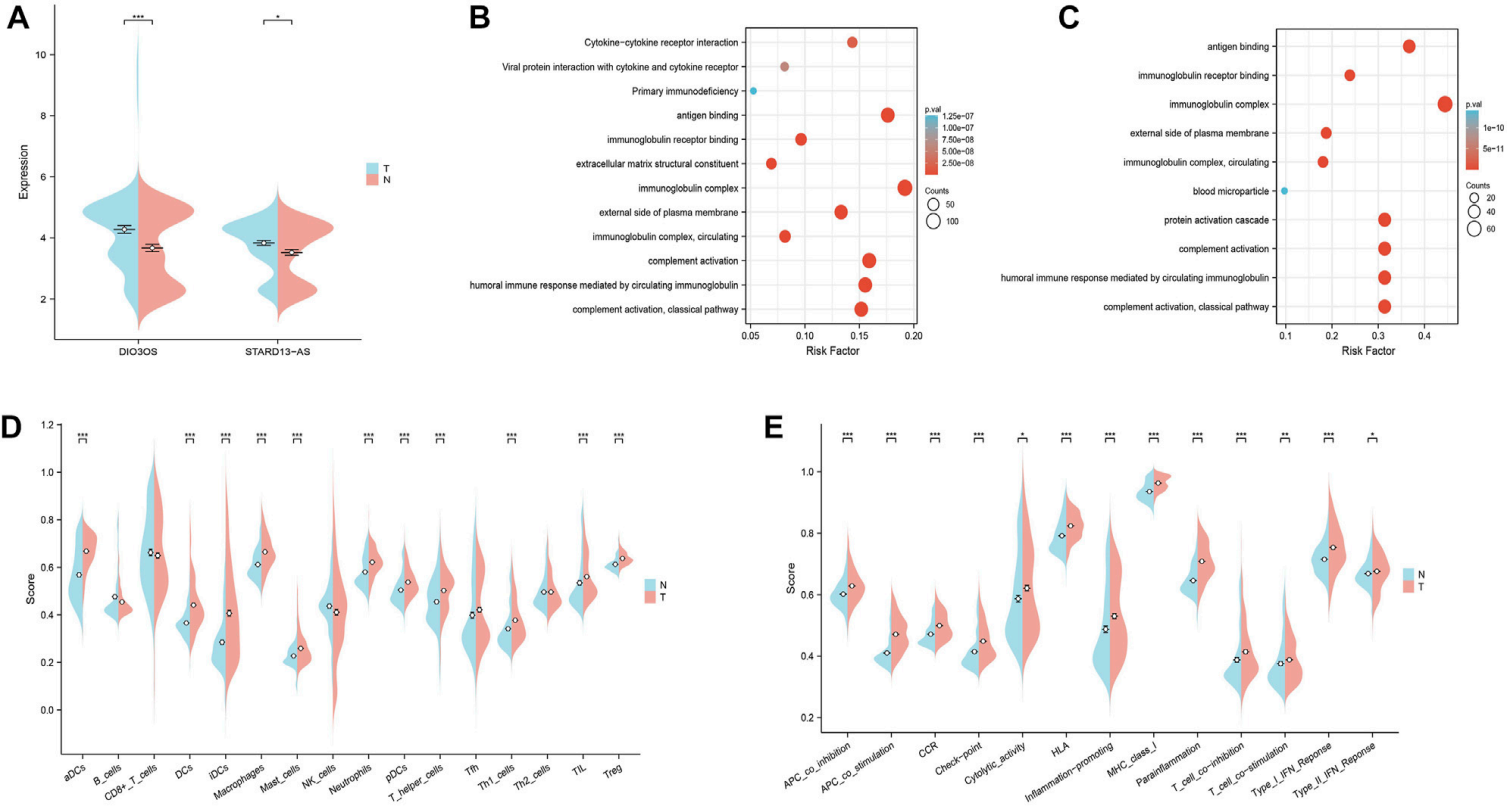
Validate the LncRNAs associated with cuproptosis in GEO databases in the tumor and non-tumor groups. **(A)** DIO3OS and STARD13-AS expression levels in tumor and non-tumor groups. **(B)** GO and KEGG analysis of DIO3OS. **(C)** GO and KEGG analysis of STARD13-AS. **(D)** The ssGSEA scores of immune cells **(E)** The ssGSEA scores of immune-related functions.

**FIGURE 14:**
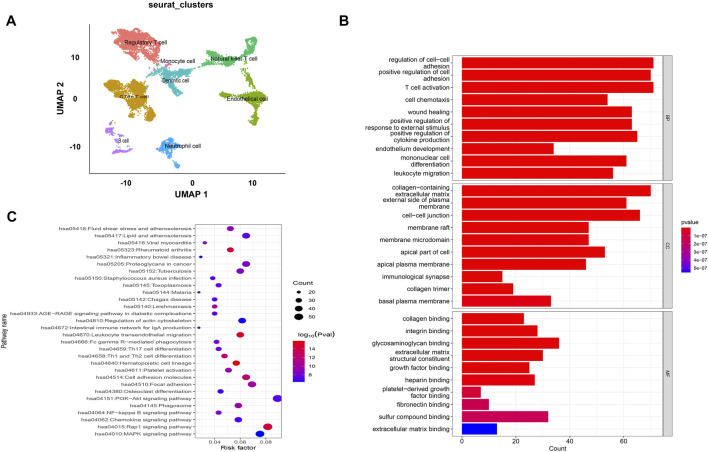
Validate the LncRNAs associated with cuproptosis in GSE191288. **(A)** UMAP plot of the analysed single cells. **(B,C)** GO and KEGG analysis of cell marker genes.

The GEO datasets were additionally utilized to investigate the relationship between cuproptosis-related LncRNA expression levels and immunological responses. DIO3OS and STARD13-AS were observed to be overexpressed in the tumor group (Figure 13A). GO and KEGG analysis revealed potential mechanisms of DIO3OS and STARD13-AS related to the immune response, protein creation, inflammatory response, cell phagocytosis, and recognition (Figures 13B, C). The immune infiltration analysis revealed significant differences in the inflammatory response, inflammation promotion, ATC co-stimulation, T cell co-stimulation, and CCR between the tumor and non-tumor groups (*p* < 0.05). Mast cells, DCs, macrophages, neutrophils, and T helper cells all significantly increased in the tumor group (*p* < 0.05). This is in accordance with prior results that these lncRNAs are linked to immunological responses (Figures 13D, E). Furthermore, the scRNA-seq data in the GSE191288 dataset were quality-controlled and preprocessed, and UMAP was used to show the high-dimensional scRNA-seq data before Cellmarker was used to identify the cell type. Single-cell gene expression profiles reveal seven major cell types in the THCA: regulatory T cells, CD8^+^ T cells, monocytes, B cells, dendritic cells, endothelial cells, and neutrophils. Numerous cancer-related pathways were upregulated in the thyroid tissue; including T cell activation, immune response activation, and immune response-activating signal transduction, among others (Figure 14).

## 4 Discussion

THCA is the most prevalent and rapidly increasing malignant tumor of the endocrine system. Despite a higher overall survival rate than other malignancies, there was a difference in the 5-year relative survival rate of patients with THCA between China and the United States (Miller et al., 2019). The pathophysiology of THCA remains incompletely understood and requires a multimodal treatment approach. Accurately predicting the prognosis and survival of patients with THCA is crucial for preventing and managing the disease. This requires the development of trustworthy THCA risk profiles. The non-protein-coding RNA family, known as lncRNA, has attracted much attention in recent years (Tan et al., 2021). Numerous studies have revealed that lncRNAs may play a significant role in tumorigenesis and development, the immune system, and inflammatory gene expression, making it one of the key targets for the treatment of THCA. The emergence of modern genomics and bioinformatics has made it possible to uncover the role of lncRNA in THCA, supported by the gradual maturation of detection technologies (Kong et al., 2019; Wang et al., 2019; Tang D et al., 2022). According to Pang et al.'s study, lncRNA DUXAP8 was significantly expressed in THCA, enabling it to serve as a target of miR-20b-5p. This interaction regulated the expression of proteins such as SOS1, c-myc, and CCND1, consequently inhibiting abnormal proliferation of thyroid cancer cells (Pang and Yang, 2021). Additionally, the knockdown of LINC00311 has been shown to suppress the development, proliferation, migration, and invasion of spheroids in THCA cells *in vitro* through the miR-330-5p/TLR4 pathway (Gao et al., 2020). Cuproptosis is a distinct type of cell death caused by excessive intracellular copper accumulation, which results from FDX1-mediated mitochondrial protein toxicity stress dependent on the mitochondrial TCA cycle (Duan and He, 2022; Tang R et al., 2022). Modulating intracellular copper levels and oxidase activity can partially mitigate mitochondrial respiratory deficits, but these solutions require further clinical testing (Ghosh et al., 2014). Nevertheless, the precise mechanism of action of cuproptosis-related lncRNAs and their relationship with THCA requires further research.

Seven lncRNAs associated with cuproptosis were identified as prognostic factors for THCA in this study, including AC108704.1, DIO3OS, AL157388.1, AL138767.3, STARD13-AS, AC008532.1, and PLBD1-AS1. By applying co-expression, univariate, and multivariate Cox analyses, the study found that these prognostic features were independent of other typical clinical features. Using ROC curves, survival analysis, and line maps, the high- and low-risk groups of THCA patients could be accurately predicted based on their risk scores, demonstrating that these lncRNAs were reliable prognostic factors. Moreover, the study showed that the prediction model based on cuproptosis-related lncRNAs can accurately predict the prognosis of THCA patients, considering factors such as age, stage, and risk score.

The possibility of DIO3OS as a biomarker for hepatocellular, lung, and pancreatic malignancies has been demonstrated (Cui et al., 2019; Wang et al., 2020; Zhang M et al., 2021). Inhibiting DIO3OS expression by blocking the DIO3OS/let-7d/NF-B2 axis, which lowers the expression of ki-67 and PCNA and reduces cancer cell viability (Wang et al., 2021). Triiodothyronine (T3) insufficiency results from increased deiodinase 3 (D3) activity caused by DIO3OS inactivation, which affects metabolic function by altering thyroid hormone signaling pathway (Chen et al., 2021). DIO3OS, as a cis-transcription element, can influence the expression of nearby genes at the transcription site. Additionally, it can target miR-18a-3p, miR-1913, and miR-266-3p to exert chromatin localization function and obstruct protein binding in the DNA region (Chen et al., 2021). AL138767.3 has been identified as a prognostic biomarker for glioma instability (Maimaiti et al., 2021). A 645 bp non-coding RNA (ncRNA) named STARD13-AS found on chromosome 13q13.1 was connected to numerous tumor progressions. RNA binding proteins such as NANOG, OTX2, POU5F1, SOX17, TBXT, and others can directly interact with STARD13-AS, which is highly expressed in enterocyte progenitor cells. It may also facilitate the interaction of the cohesins or the enhancer and mediator complex (Zhang and Chambers, 2019; Zhang et al., 2021). When STARD13-AS expression was controlled, LoVo and SW620 cells expressed higher levels of E-cadherin and N-cadherin, which reduced the growth and spread of colorectal cancer cells (Nasrallah et al., 2014; Yang et al., 2019). By blocking miR-9-5p, STARD13-AS inhibits the proliferation and migration of prostate cancer cells, thereby slowing tumor growth (Chen et al., 2019). STARD13-AS may engage in the TCA cycle to regulate proteins involved in mitochondrial activity and energy substrate oxidation through a process similar to cuproptosis by inhibiting miR-19a-3p (Pinto et al., 2017). As an autophagy-related lncRNA, PLBD1-AS1 has been demonstrated to be significantly correlated with the prognosis of hepatocellular carcinoma and may be linked to TP53BP1 and CHMP4B (Deng et al., 2021). PLBD1-AS is overexpressed in breast and liver cancer cells, which is mostly accomplished by physiological and pathological processes, such as cell metabolism, cell proliferation, apoptosis, and immune response (Luo et al., 2022; Safarzadeh et al., 2022). While these lncRNAs have been demonstrated to impact the development of various cancers, further research is necessary to determine their specific effects on THCA. There is no evidence to suggest that AC108704.1, AL157388.1, and AC008532.1 have any involvement in cancer.

According to the PCA results, seven cuproptosis-related lncRNAs could discriminate between groups at high and low risk. Additionally, GO and KEGG analyses revealed that, based on biological processes, such as protein synthesis, transport, angiogenesis, apoptosis, enzyme regulation, and immune response, cuproptosis-related lncRNAs may be directly related to THCA formation through the TGF-β and PISK-Akt signaling pathways. By verifying GEO databases, the relationship between lncRNA and THCA was further confirmed. Several studies have highlighted the significance of GDF6, RGS4, FGF7, GLIS1, SST, and MT3 in predicting patient prognosis (MacDonald et al., 2020; Puig-Domingo et al., 2014; Nikiforova et al., 2019; Ji et al., 2020; Fan et al., 2022; Li et al., 2022). DPP4 facilitates epithelial-to-mesenchymal transformation and thyroid cancer cell metastasis by interacting with α4 and β1 integrin subunits to activate the transcription of the TGFB1 protein via the FAK/AKT/C-JUN/TGF-β signaling pathway (He et al., 2022). Moreover, the regulation of thyroid cancer cell proliferation and tumor growth can be achieved through the competition for miR-34a binding via the PI3K/AKT signaling pathway by downregulating miR-34a levels downstream of the hepatocyte growth factor receptor proteins by reducing lncRNA XIST expression (Liu et al., 2018).

During tumor development, immune cell infiltration in the tumor microenvironment changes. The relationship between immune-related functions, TMB, and risk ratings in patients with THCA was investigated. Various studies have linked T-cell follicular helper cells, T-cell regulation, and B-cell memory to adverse effects in THCA (Koida et al., 2007; Xie et al., 2020; Wang et al., 2022). Single-cell analysis of GEO databases has revealed that regulatory T cells, CD8^+^ T cells, monocytes, B cells, dendritic cells, and neutrophils are crucial components of THCA tissue. Additionally, tumor metastasis frequently occurs in lymph nodes. By controlling the invasion and response of immune cells, the interaction between tumor invasion-related cells and the host immune system is achieved (Garnier et al., 2019).

The high-risk group exhibited higher expression levels of B cells, dendritic cells, T helper cells, Th1 helper cells, and tumor-infiltrating lymphocytes, as indicated by the results of immune infiltration analysis. Given that this result is highly consistent with previous findings, it is likely that the high-risk group will have a poorer prognosis than the low-risk group. These results suggest that cuproptosis, especially macrophages and T cells, may have a significant role in regulating the tumor microenvironment. In addition, the ssGSEA score revealed that patients in the high-risk group exhibited more pronounced immunological traits, including antigen presentation, immune response, para-inflammatory response, and T cell response, all of which are associated with the THCA tumor immune microenvironment and immune response suppression. The high-risk group had higher immunoscores and lower tumor purity scores compared to the low-risk group, as indicated by the estimated, immune, and stromal scores.

**FIGURE 15:**
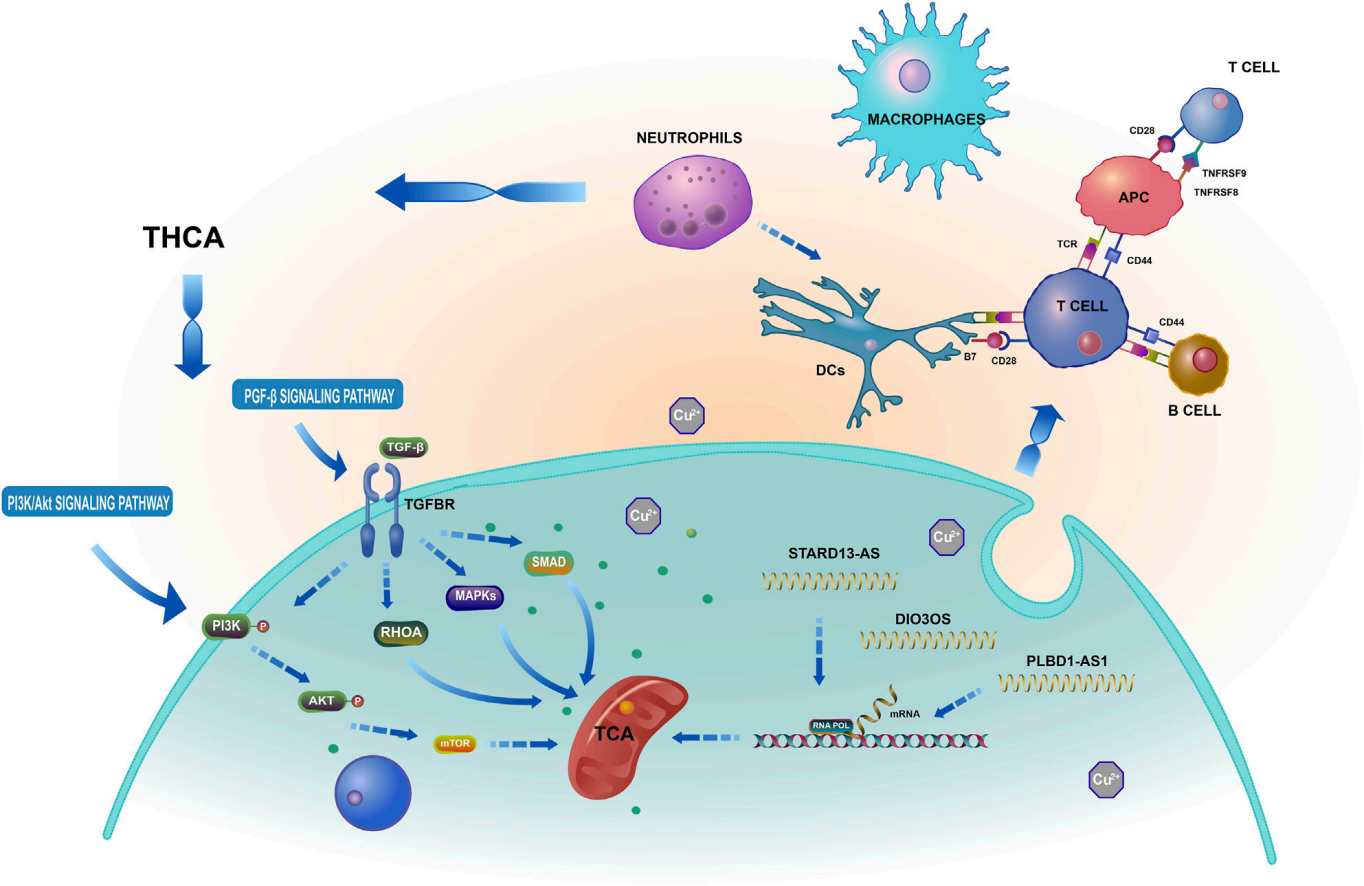
Based on a bioinformatics analysis that the possible mechanism of cuproptosis-related lncRNAs influence THCA.

By preventing T cell expression, LAG3 and CTLA4 play crucial roles in the immunological escape mechanism of THCA cells (Park et al., 2022). CD80, CD86, and CD28 can elicit immunosuppression by preventing B and T cell activation and proliferation. Nevertheless, this requires the cooperative action of TNFRSF18, TNFRSF8, and BTLA (Wu et al., 2021). Interestingly, the ICOS, LAG3, IDO2, TNFRSF18, CTLA4, CD80, and BTLA groups exhibited superior performance in the low-risk group than in the high-risk group. Using the TIDE algorithm to assess the clinical response of THCA patients to immune checkpoint inhibitor (ICI) treatment, we found that the TIDE score of the high-risk group was significantly higher than that of the low-risk group. This higher score indicated a higher probability of immune escape and limited response to ICI treatment, leading to a shorter survival time for patients in this group. Based on the aforementioned results, we suggest that cuproptosis-related lncRNAs are involved in several crucial physiological processes, dependent on multiple targets and pathways, and closely related to THCA tumor immunity, as shown in Figure 15. A follow-up experimental study is required to comprehend better the connection between cuproptosis-related lncRNAs and immunological performance.

Finally, we investigated the drugs’ sensitivity and used the pRRophetic package to identify effective drugs for THCA immunotherapy. Clinical trials have demonstrated that the EGFR inhibitor, lapatinib, can modestly increase the survival time of breast cancer patients and exhibit antitumor efficacy (Goss et al., 2013). Lapatinib can reduce THCA cell proliferation and improve medication sensitivity by controlling the phosphorylation of ERK and AKT (Ohno et al., 2022). Dasatinib, by activating Src signaling, c-Src, and Lyn proteins, causes cell cycle arrest and cell death in THCA fractions and acts as an anti-THCA equilibrium agent (Chan et al., 2012). However, there is insufficient evidence in the current study to determine whether these anticancer drugs are more beneficial for the low- or high-risk groups, and further research is required to identify their precise mechanisms.

## 5 Conclusion

The study successfully identified clinical characteristics that affect THCA patients’ prognosis and established a predictive risk model that categorizes patients into low- and high-risk groups based on cuproptosis-related lncRNA. The prognostic model showed a strong association with THCA prognosis. The correlation between THCA and immunological response, inflammatory response, cell proliferation, *etc.*, was shown by immune infiltration, GO, and KEGG assays based on cuproptosis-related lncRNAs. However, this study has some limitations, although it contributes new theoretical research approaches to investigating the process of cuproptosis and gaining new insights into clinical practice for THCA patients. The lack of survival prognosis information on cuproptosis-related lncRNAs in the GEO dataset made it challenging to gather additional data to support the prognostic model. The validity of cuproptosis-related lncRNAs needs to be confirmed through comprehensive clinical trials since the study’s findings are based on publicly available databases, and there was a lack of data on clinical samples, leaving the subject still unclear. Nonetheless, we discovered a potential link between cuproptosis-related lncRNA and THCA that could serve as a basis for further *in vitro* and clinical experiments to validate the association.

## Data Availability

The raw data supporting the conclusion of this article will be made available by the authors, without undue reservation.

## Supplementary material

SUPPLEMENTARY TABLE S1 The study's THCA patients' clinical characteristics.
