# Supplementary figures and images for: Cuproptosis-related lncRNAs predict prognosis and immune response of thyroid carcinoma
Source: Front Genet. 2023 Jul 4;14:1100909. doi: 10.3389/fgene.2023.1100909 (PMC10352785; doi:10.3389/fgene.2023.1100909)

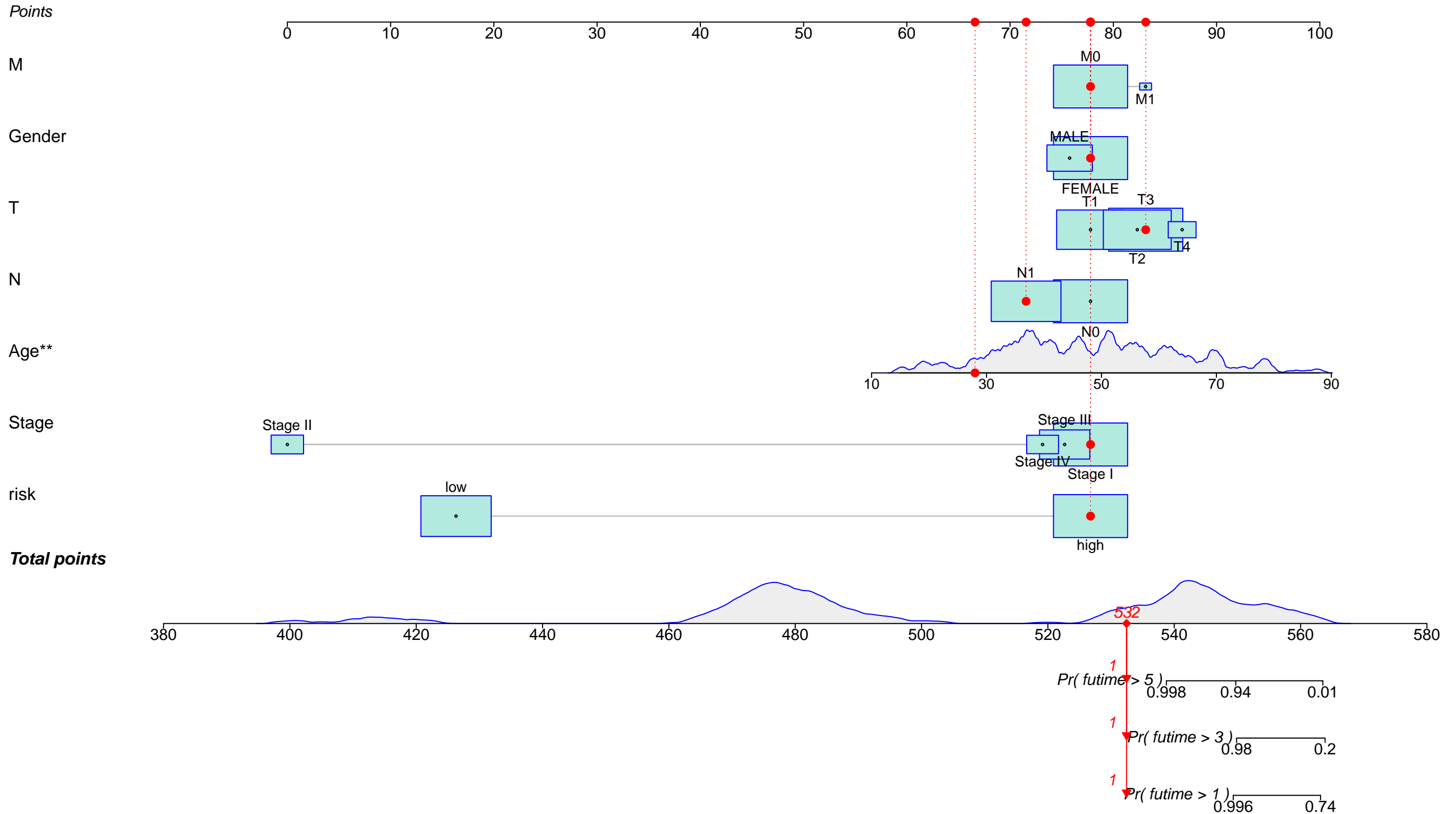

Supplement: Supplementary file 1 [file DataSheet2.PDF]

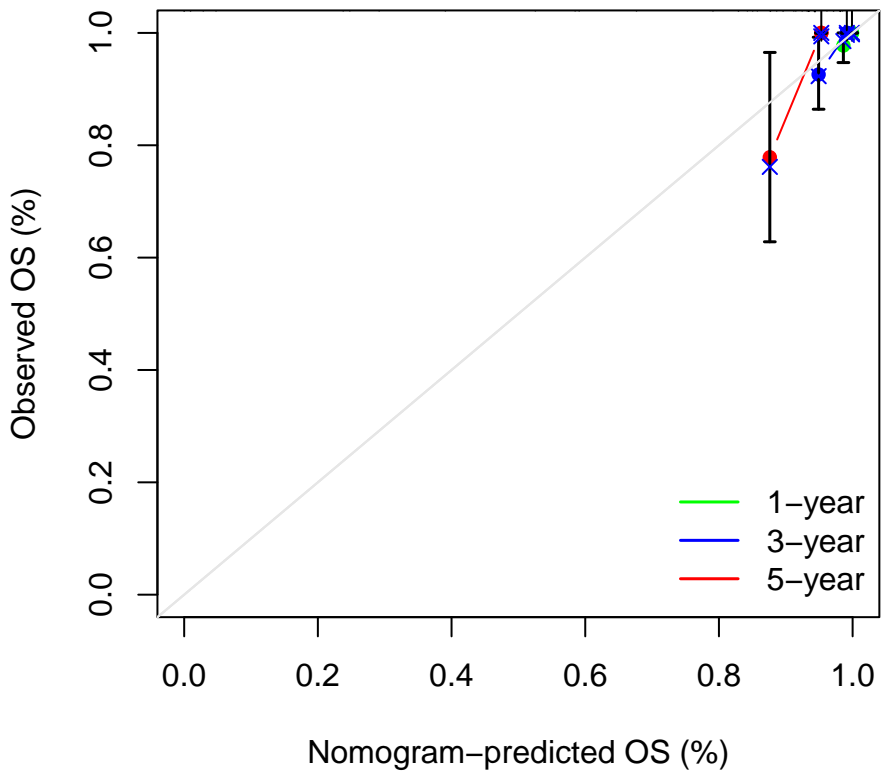

Supplement: Supplementary file 2 [file DataSheet3.PDF]

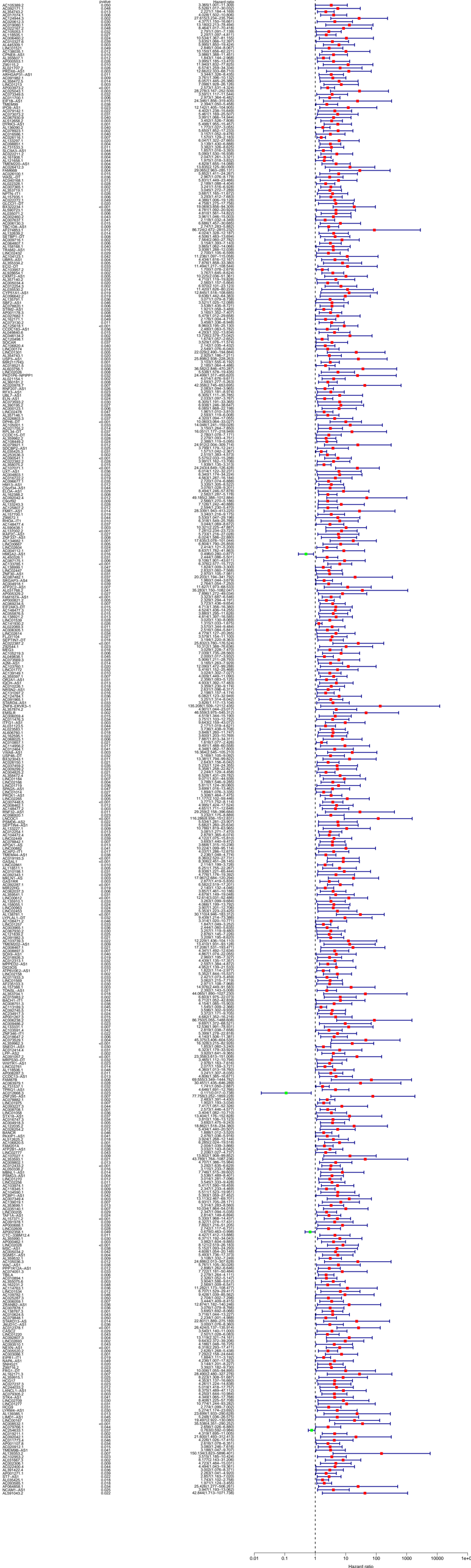

Supplement: Supplementary file 3 [file DataSheet1.PDF]
